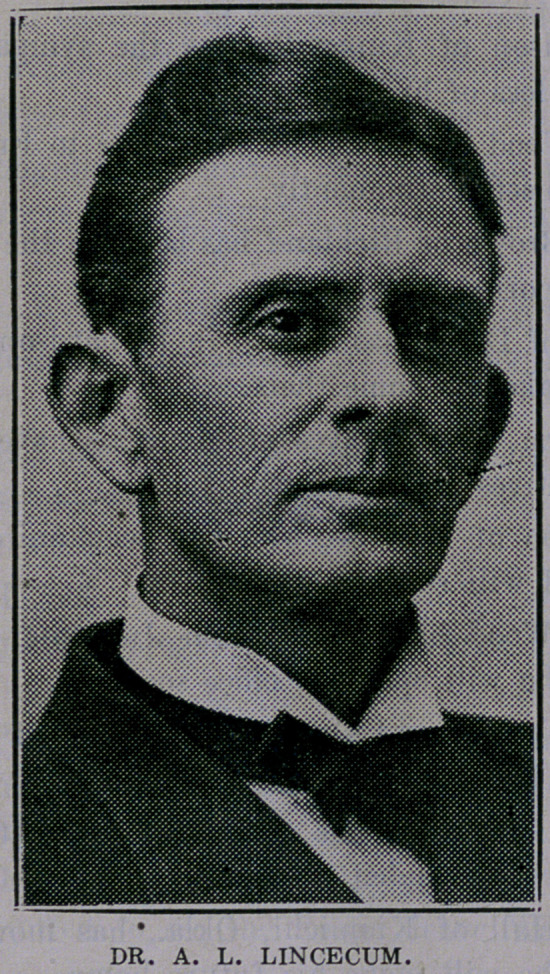# The New State Health Officer

**Published:** 1915-02

**Authors:** 


### The New State Health Officer.

Dr. W. B. Collins, of Lovelady, Texas, has been appointed by Governor Ferguson to succeed Dr. Steiner as State Health Officer.

Dr. Collins is one of the best known physicians of East Texas. He graduated with first honors from the Kentucky School of Medicine in 1885, and in 1887 was given an honorary diploma from Louisville Medical College. He has served his profession as president of the county society in which he lived, as president of the East Texas Medico-Chirurgical Society, which was later reorganized into the district medical society, and as a member of the State Examining Board he has done excellent work for the past seven years, having been appointed when the Board was first formed.

The following resolution, which was passed by his colleagues, shows in what esteem he is held by them:

"Resolved, That we hereby express our sincere appreciation for the valuable services Dr. Collins has hendered the State Board of Medical Examiners; for his conscientious and impartial stand upon every question involving equity and justice, and that we most heartily endorse his appointment to the responsible position of State Health Officer. We can commend to the profession of Texas Dr. Collins as a man fully equipped from every standpoint to discharge the duties of the office of State Health Officer. We commend him to the profession as a man who is willing to co-operate with them upon every question affecting the advancement of the Department of Public Health, and as a man who will meet them on half-way ground on any proposition. We commend him to the citizenship of Texas as a citizen above reproach and as a conscientious, progressive medical men, whose sole aim and ambition will be to faithfully discharge the duties of the office. We earnestly ask for Dr. Collins the co-operation of both the profession and citizenship of Texas. We especially commend him to the various

DR. W. B. COLLINS.

women's organizations, clubs, etc., as a man who will be willing to give a listening ear to any reasonable proposition, and one who is not only willing but anxious upon any and all occasions to do anything within his power to advance any laudible enterprises advocated by said organizations or clubs."

Dr. Collins is a prominent member of the State Medical Society, and his appointment is pleasing to the physicians of the State, who will no doubt give him their earnest co-operation.

---

Dr. W. A. Davis, the newly appointed Registrar of Vital Statistics, was born November 11, 1875. He received his education in the public schools, the State University, and the Medical Department at Galveston. The doctor graduated in medicine in the Missouri Medical College at St. Louis in 1899.

Dr. Davis was superintendent of the Monterey Hospital for three years and for three years he was county physician in Atascosa county. He is a member of the county, State and American Medical Association.

---

The appointment of Dr. A. L. Lincecum of El Campo as Assistant State Health Officer is timely. The doctor is a student of men and affairs, and Dr. Collins is to be congratulated upon having such an efficient associate in his work. Dr. Lincecum's numerous friends

DR. A. L. LINCECUM.

are much pleased with the honor that has been conferred upon him. Let us all drink a toast (of good sterilized water, of course) to the new State Health Department and pledge them our co-operation for a "healthier Texas."

---

The following doctors have been appointed to serve on the Board of Medical Examiners of the State: Dr. M. B. McElhanan, Secretary-Treasurer, Belton; Dr. John McCelvey, Temple; Dr. W. P. Swain, Dallas; Dr. H. B. Mason, Temple; Dr. S. L. Scothorn, Dallas; Dr. H. C. Morrow, Austin; Dr. T. J. Crow, President, Dallas; Dr. M. A. Cooper, Childress; Dr. M. F. Bettencourt, Mart; Dr. J. J. Williams, Limestone county; Dr. J. H. McLean, Fort Worth.

## Figures and Tables

**Figure f1:**
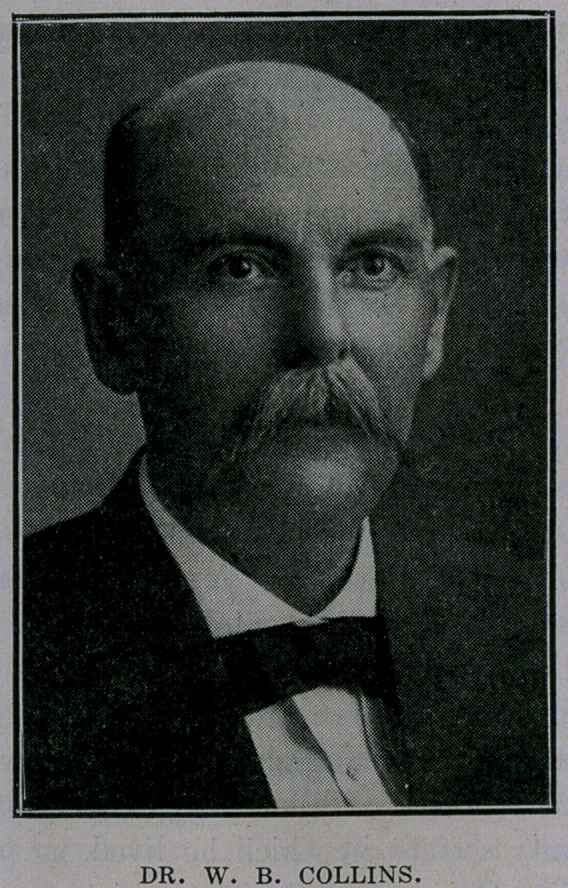


**Figure f2:**